# Successful surgical repair of a huge left ventricular pseudoaneurysm after repair of left ventricular rupture during mitral valve replacement

**DOI:** 10.1093/jscr/rjae636

**Published:** 2024-10-07

**Authors:** Kyoka Hayashi, Ryohei Ushioda, Jun Maruoka, Kaname Shimizu, Kentaro Shirakura, Nobuhiro Mochizuki, Yuki Setogawa, Ryo Okubo, Miyamoto Hiroyuki, Shougo Takahashi, Shingo Kunioka, Masahiro Tsutsui, Kamiya Hiroyuki

**Affiliations:** Department of Cardiac Surgery, Asahikawa Medical University, Asahikawa, Japan; Department of Cardiac Surgery, Asahikawa Medical University, Asahikawa, Japan; Department of Cardiac Surgery, Asahikawa Medical University, Asahikawa, Japan; Department of Cardiac Surgery, Asahikawa Medical University, Asahikawa, Japan; Department of Cardiac Surgery, Asahikawa Medical University, Asahikawa, Japan; Department of Cardiac Surgery, Asahikawa Medical University, Asahikawa, Japan; Department of Cardiac Surgery, Asahikawa Medical University, Asahikawa, Japan; Department of Cardiac Surgery, Asahikawa Medical University, Asahikawa, Japan; Department of Cardiac Surgery, Asahikawa Medical University, Asahikawa, Japan; Department of Cardiac Surgery, Asahikawa Medical University, Asahikawa, Japan; Department of Cardiac Surgery, Asahikawa Medical University, Asahikawa, Japan; Department of Cardiac Surgery, Asahikawa Medical University, Asahikawa, Japan; Department of Cardiac Surgery, Asahikawa Medical University, Asahikawa, Japan

**Keywords:** mitral valve replacement, left ventricular rupture, left ventricular pseudoaneurysm

## Abstract

A 78-year-old man underwent pericardial patch repair for left ventricular (LV) rupture during mitral valve replacement. After the first operation, a huge (>10 cm) LV pseudoaneurysm was detected, necessitating reoperation. LV rupture is a rare but often fatal complication of mitral valve replacement. Although repair of LV rupture during mitral valve replacement has been reported, the development of pseudoaneurysm after such repair is exceedingly rare. In this case, we successfully treated a huge LV pseudoaneurysm using two pericardial patches to sandwich the rupture hole from the inside.

## Introduction

Left ventricular (LV) rupture is a rare and fatal complication following mitral valve replacement (MVR), occurring in 0.5%–2.0% of cases [1] and having an extremely high mortality rate of 65%–75%, according to Deniz *et al*. [2]. Given the few available reports [3, 4], pseudoaneurysm after LV rupture following MVR is rare and has no established repair methods. Here, we presented a case of successful surgical repair of a huge LV pseudoaneurysm after LV rupture during MVR.

## Case report

A 78-year-old man was admitted to our hospital because of infectious endocarditis. His underlying diseases were hypertension, dyslipidemia, and diabetes.

Laboratory data revealed anemia (hemoglobin 10.8 g/dl); mild renal dysfunction (blood urea nitrogen 31.0 mg/dl, creatinine 1.62 mg/dl); and elevated inflammatory markers (white blood cell 22 350/μl, C-reactive protein 12.6 mg/dl). Transthoracic echocardiography (TTE) revealed an ejection fraction of 68% and a mobile 11 × 33-mm vegetation on the posterior apex of the mitral valve. Emergency surgery was performed because of the risk of embolization.

**Figure 1:**
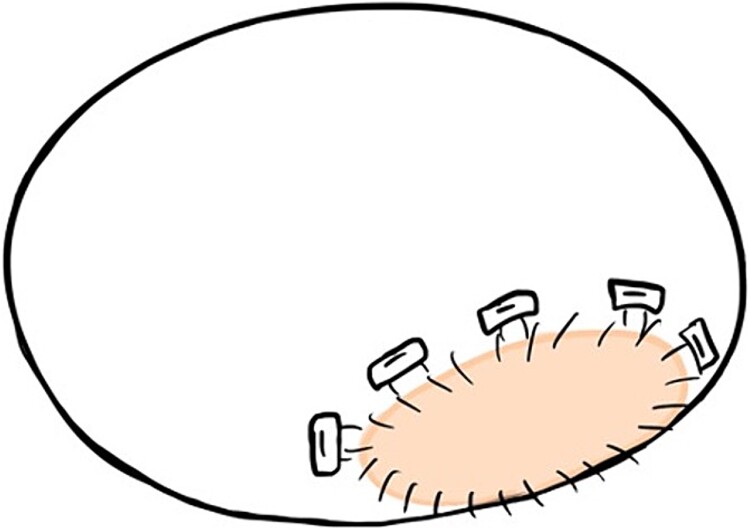
The 2 × 4-cm pericardial patch is seen to cover the P2 site of the annulus from the inside during the first operation.

During the first operation, an abscess was discovered in the posterior mitral valve annulus. After removing the vegetation, the mitral valve was replaced (Epic 27 mm; St. Jude Medical, Saint Paul, MN, USA), using the flip-over technique, with the anterior leaflet tissue as reinforcement for the posterior annulus side. Upon cardiopulmonary bypass weaning, bleeding occurred from the posterior surface of the inferior vena cava secondary to LV rupture. Sequentially, the tissue valve was removed, the P2 site of the annulus was covered with a 2 × 4-cm pericardial patch from the inside (Fig. 1), and valve replacement was repeated. The patient recovered without complications and was transferred to another hospital on postoperative Day 39.

**Figure 2:**
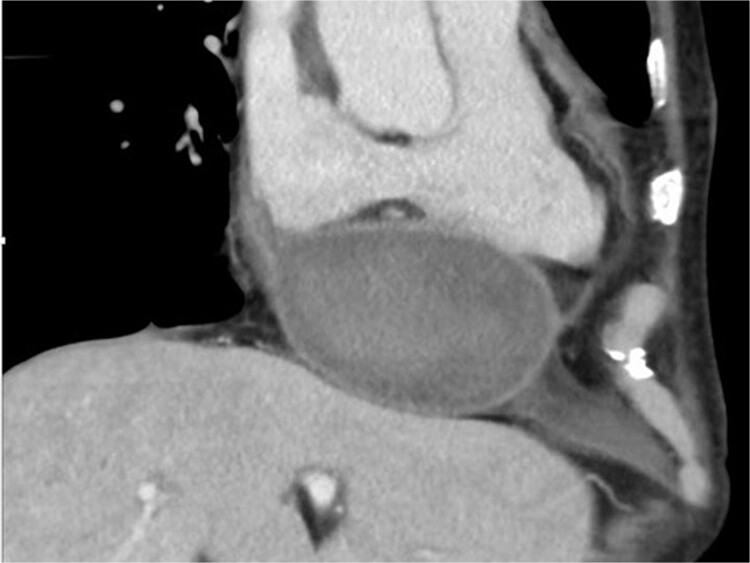
Computed tomography scan at 2 months after left ventricular rupture repair. There is a huge pseudoaneurysm (110 × 57 mm) on the posterior side of the left ventricle.

Two months later, follow-up computed tomography revealed a huge pseudoaneurysm (110 × 57 mm) on the posterior side of the LV (Fig. 2). Based on TTE confirmation of blood flow into the pseudoaneurysm, a second operation was performed because of the high risk of cardiac rupture.

**Figure 3:**
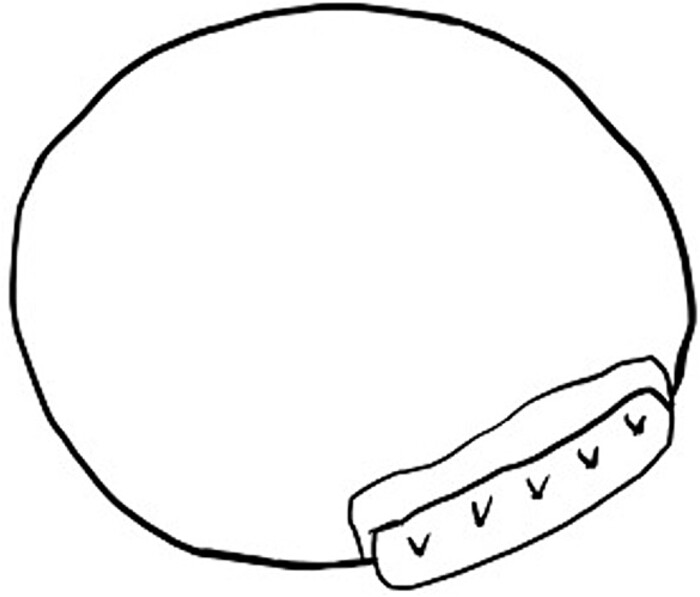
The two pericardial patches are used to close a 5-mm rupture hole under the posterior mitral annulus.

Intraoperatively, removal of the hematoma revealed that the pseudoaneurysm was secondary to LV rupture. The previously implanted pericardial patch broke, exposing a 5-mm rupture hole on the posterior mitral annulus. Thereafter, two pericardial patches were used to close the rupture from the inside (Fig. 3), and a repeat MVR (Epic 27 mm; St. Jude Medical, Saint Paul, MN, USA) was performed. The postoperative course was uneventful, and the patient was discharged 22 days later. One year after the second surgery, the patient has remained alive without cardiac events.

## Discussion

Although there have been reports on repair of LV rupture during MVR [5, 6], cases of subsequent pseudoaneurysm development are rare. This was a unique case of a huge LV pseudoaneurysm that was successfully treated using valve annuloplasty and two pericardial bands to close the rupture hole from the inside. The surgical methods for LV rupture repair remain debatable. There can be a significant discrepancy between the epicardial bleeding site and the endocardial rupture site [7]. Previous studies have suggested that using a pericardial patch to cover a large area, including the rupture site, can be an effective strategy [8]. In this case, we initially employed this strategy to widely cover the rupture hole from the inside. However, the pericardial patch detached, leading to the development of a pseudoaneurysm due to mild edema and fragility of the LV myocardium. During the second operation, the same rupture hole was identified from the endocardial side and was repaired by sandwiching between two pericardial patches, along with mitral valve annulus reinforcement with a pericardial patch. The close proximity of the LV rupture to the mitral annulus suggested that the pressure exerted on the valve may have been transferred to and caused detachment of the previous patch [9]. Given the complexity of this rupture, the aforementioned technique may be a valuable approach for such cases.

We successfully treated a huge LV pseudoaneurysm after LV rupture using two pericardial bands to close the rupture hole from the inside. Although the long-term durability of this approach requires further follow-up, this patching technique shows promise as a valuable method for repairing LV rupture around the mitral annulus.
